# Advances and Perspectives in the Treatment of T-PLL

**DOI:** 10.1007/s11899-020-00566-5

**Published:** 2020-02-07

**Authors:** Till Braun, Jana von Jan, Linus Wahnschaffe, Marco Herling

**Affiliations:** 1 Department I of Internal Medicine, Center for Integrated Oncology (CIO), Aachen-Bonn-Cologne-Duesseldorf, University of Cologne (UoC), 50937 Cologne, Germany; 2 Excellence Cluster for Cellular Stress Response and Aging-Associated Diseases (CECAD), UoC, 50937 Cologne, Germany; 3 Center for Molecular Medicine Cologne (CMMC), UoC, 50937 Cologne, Germany

**Keywords:** T-PLL, T cell lymphoma, Alemtuzumab, p53 reactivation, HDAC, BCL2 antagonists, JAK/STAT inhibition

## Abstract

**Purpose of Review:**

T cell prolymphocytic leukemia (T-PLL) is a rare mature T cell tumor. Available treatment options in this aggressive disease are largely inefficient and patient outcomes are highly dissatisfactory. Current therapeutic strategies mainly employ the CD52-antibody alemtuzumab as the most active single agent. However, sustained remissions after sole alemtuzumab-based induction are exceptions. Responses after available second-line strategies are even less durable. More profound disease control or rare curative outcomes can currently only be expected after a consolidating allogeneic hematopoietic stem cell transplantation (allo-HSCT) in best first response. However, only 30–50% of patients are eligible for this procedure. Major advances in the molecular characterization of T-PLL during recent years have stimulated translational studies on potential vulnerabilities of the T-PLL cell. We summarize here the current state of “classical” treatments and critically appraise novel (pre)clinical strategies.

**Recent Findings:**

Alemtuzumab-induced first remissions, accomplished in ≈ 90% of patients, last at median ≈ 12 months. Series on allo-HSCT in T-PLL, although of very heterogeneous character, suggest a slight improvement in outcomes among transplanted patients within the past decade. Dual-action nucleosides such as bendamustine or cladribine show moderate clinical activity as single agents in the setting of relapsed or refractory disease. Induction of apoptosis via reactivation of p53 (e.g., by inhibitors of HDAC or MDM2) and targeting of its downstream pathways (i.e., BCL2 family antagonists, CDK inhibitors) are promising new approaches. Novel strategies also focus on inhibition of the JAK/STAT pathway with the first clinical data. Implementations of immune-checkpoint blockades or CAR-T cell therapy are at the stage of pre-clinical assessments of activity and feasibility.

**Summary:**

The recommended treatment strategy in T-PLL remains a successful induction by infusional alemtuzumab followed by a consolidating allo-HSCT in eligible patients. Nevertheless, long-term survivors after this “standard” comprise only 10–20%. The increasingly revealed molecular make-up of T-PLL and the tremendous expansion of approved targeted compounds in oncology represent a “never-before” opportunity to successfully tackle the voids in T-PLL. Approaches, e.g., those reinstating deficient cell death execution, show encouraging pre-clinical and first-in-human results in T-PLL, and urgently have to be transferred to systematic clinical testing.

## Introduction

T cell prolymphocytic leukemia (T-PLL) is an aggressive peripheral (post-thymic) T cell malignancy [1••]. With an incidence of ≈ 2.0/million/year, it is still the most common mature T cell leukemia in Western countries [2]. The median age at disease onset is ≈ 65 years [3]. T-PLL patients typically present with exponentially rising blood lymphocyte counts, bone marrow infiltration, splenomegaly, and small lymphadenopathy. Their dismal prognosis is reflected in a median overall survival (OS) from diagnosis of < 3 years [4–7].

T-PLL cells show a refractory behavior towards conventional chemotherapeutics like alkylating agents or CHOP polychemotherapy [8, 9]. Even after responses to the humanized CD52-antibody alemtuzumab, the most active single substance in T-PLL being effective in > 80% of patients, nearly all relapse at median within 12 months [3, 5, 10], with very limited options to salvage. Allogenic hematopoietic stem cell transplantation (allo-HSCT) is the only treatment of curative potential, however, most patients are ineligible, or experience critical side effects, or relapse within the first years from this intervention [11•]. There is currently no drug that carries FDA or EMA approval status for T-PLL. This applies to alemtuzumab as well and this antibody is currently only available through a named-patient program. These circumstances emphasize the need for new active treatments in this orphan disease.

**Fig. 1:**
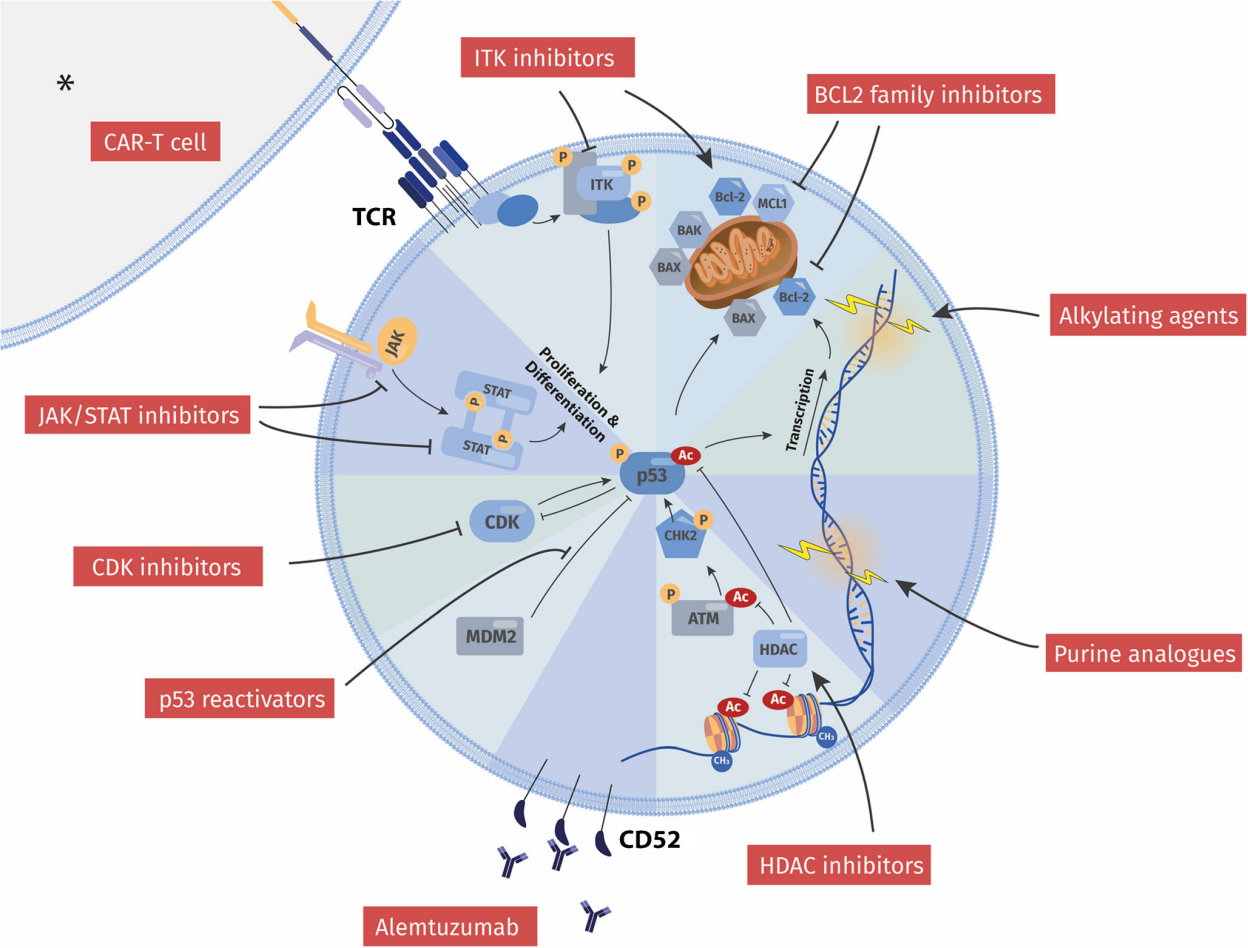
Overview of current and novel treatment strategies in T-PLL in relation to functional hallmarks of the tumor cell. Illustrated are categories of targets and modes of action of both established and most promising compound classes. Current treatment of T-PLL employs a (chemo)-immunotherapeutic approach based on alemtuzumab as well as on DNA-damaging alkylating agents and purine analogs. The inability of the T-PLL cell to evoke adequate p53-mediated responses to DNA-injuries, e.g., via enforced checkpoints or an apoptotic fate, stands at the center of its notorious therapy resistance. Novel strategies, however, revert the repressed state of functionally competent p53, or harness the apoptotically primed cellular state, or interrupt vital growth signal input. Respective examples are reactivation of p53 (e.g., via HDAC inhibitors, MDM2 inhibitors), targeting of BCL2 family members, inhibition of CDKs, or interception in JAK/STAT signal transduction. Efforts are also undertaken to utilize immunogenic cell death, e.g., by implementation of CAR-T cell therapies. * Generally, the role of cellular components of the tumor micromilieu is not established in T-PLL. Nevertheless, clinically proven GvL effects [48] and TCR-directed CAR-T cells in pre-clinical models [79••] implicate a therapeutic application of a T cell-mediated anti-T-PLL attack. Modulation of immune regulatory synapses, e.g., by perturbation of NK-cell tolerance (e.g., anti-KIR3DL2) or of macrophage inertia (e.g., SIRPαFc binding to CD47) represent interventional strategies of proven activity in other T cell tumors [76, 77], which have yet to be evaluated for their efficacy in T-PLL

Particularly in the past 5 years, modern profiling studies on sizable T-PLL cohorts and mechanistic validations have significantly advanced our disease concept. Activation of the T cell leukemia/lymphoma 1 (*TCL1*) proto-oncogenes and loss-of-function perturbations of the tumor suppressor ataxia telangiectasia mutated (*ATM*) are the most common genomic lesions of T-PLL [12•, 13••]. Both aberrations cooperate towards an aberrant DNA damage response [13••]. JAK/STAT-activation and epigenetic alterations have also emerged as hallmarks and further contribute to the genome-instability and cell-death resistant phenotype [13••, 14, 15•]. They provide new possibilities for targeting T-PLL [16].

The improved molecular understanding of T-PLL has resulted in encouraging preliminary results on the anti-leukemic activity of new substances with realistic potential to become part of our armentarium in this disease. In vitro drug screenings identified p53 reactivators, histone deacetylase (HDAC) inhibitors, B cell lymphoma 2 (BCL2) inhibitors, Janus kinase (JAK) inhibitors, and cyclin-dependent kinase inhibitors (CDK) as the most prominent categories [17, 18••]. The clinical activity of JAK/STAT inhibitors as well as of BCL2 inhibition was presented in first reports in relapsed T-PLL patients [19, 20].

Taken together, identification of actionable vulnerabilities and proof-of-principle preclinical data have initiated a dynamic era of clinical investigations of substances (re)purposed for targeting T-PLL. This systematic overview outlines the current state-of-the-art, summarizes advances in novel targeted approaches, and highlights promising directions in the treatment of T-PLL.

## Current Treatment Options

A stable or slowly progressing disease can be referred to as “inactive” T-PLL, which is initially found in ≈ 20–30% of patients [1••]. For these, there is yet no evidence for a benefit of immediate treatment initiation. However, virtually all cases convert into an “active” T-PLL disease within 1–2 years, eventually requiring treatment [21]. Currently, the recommended first-line therapy is infusional alemtuzumab followed by an allo-HSCT, where possible [5]. For transplant-ineligible patients, there is no concept of consolidation after alemtuzumab-based induction. In the following, we discuss advances in the categories of chemo(immuno)therapy and HSCT in T-PLL. Table 1 summarizes the most important clinical studies therein. Overall, the rarity of T-PLL dictates the scarcity of systematic trials. Most of them conceptualized the use of alemtuzumab, as a single agent or in combination with a chemotherapy component.

**Table 1:** Synopsis of important clinical trials that specifically addressed T-PLL

Summary of most relevant clinical studies on chemo-/immunotherapy in T-PLL									
Regime	Study design	Treatment status	*n*	ORR	CR	PR	PFS	OS	Reference
				[%]	[%]	[%]	[mo]	[mo]	
Pentostatin	Single center, retrospective	Pretreated	56	45	9	36	6	9	[9]
Alemtuzumab, iv	Single center, retrospective	Pretreated	15	73	60	13	6	8	[34]
Alemtuzumab, iv	Multicenter, prospective	Pretreated	39	76	60	16	7	10	[33]
Alemtuzumab, iv	Multicenter, retrospective	Untreated	4	75	75	0	5	8.7	[30]
		Pretreated	72	50	37.5	12.5	4.5	7.5	
Pentostatin + alemtuzumab, iv	Single center, prospective	Pretreated	13	69	62	8	7.8	10.2	[39]
Alemtuzumab, iv	Single center, prospective	Untreated	32	91	81	10	12	48	[10]
		Pretreated	45	74	60	14	12	48	
Alemtuzumab, sc		Untreated	9	33	33	0	12	48	
FMC + alemtuzumab, iv	Multicenter, prospective	Untreated	16	92	48	44	11.5	17.1	[5]
		Pretreated	9						
Bendamustine	Multicenter, retrospective	Untreated	6	55.3	20	33.3	5	8.7	[24]
		Pretreated	9						
Alemtuzumab, iv	Single center, retrospective	Untreated	13	n.a.	n.a.	n.a.	n.a.	40.5	[32]
Alemtuzumab, sc	Single center, retrospective	Pretreated	5	n.a.	n.a.	n.a.	n.a.	13.7	[32]
Alemtuzumab, iv + cladribine +/− HDAC inhibition	Single center, retrospective	Untreated	4	100	75	25	6.3	14.8	[52]
		Pretreated	4	100	100	0	11.35	23.7	
Alemtuzumab, iv	Single center, retrospective	Untreated	42	81	61	20	11	15	[31•]
		Pretreated	15	46	46	0	3	15	
Alemtuzumab, iv + pentostatin		Untreated	13	82	73	9	4.3	10.4	
		Pretreated	5	75	50	25	2.6	2.6	
FMC + alemtuzumab, sc	Multicenter, prospective	Untreated	13	68.7	32.1	36.6	7.5	11.5	[26•]
		Pretreated	5						

*iv* intravenous, *sc* subcutaneous, *FMC* fludarabine, mitoxantrone, cyclophosphamide, *HDAC* histone deacetylase

### Conventional Cytostatics

Initial, mostly futile, attempts to treat T-PLL focused on alkylators, anthracyclines, and purine analogs or their combinations. CHOP (cyclophosphamide, doxorubicin, vincristine, prednisolone) or CHOP-like regimens induced responses in only 33% of previously untreated T-PLL without adding to the median overall survival (OS) of 7.5 months that patients faced during this era [8].

Treatment with the purine analogs pentostatin and nelarabine showed overall response rates (ORRs) in previously untreated T-PLL of 33–45% and 20%, respectively, with 63% for the combination of nelarabine with fludarabine. However, complete responses (CRs) were rarely accomplished in these studies and the remissions where short-lived (≈ 6 months) [9, 22, 23]. Bendamustine showed an ORR of 67% in treatment-naive T-PLL and of 53% in a mixed cohort (6 untreated and 9 previously treated), presenting the most promising results of a single chemotherapeutic with a milder reported toxicity profile compared to other cytostatics in T-PLL [24, 25•].

Polychemotherapy of fludarabine, mitoxantrone, and cyclophosphamide (FMC) induced high ORRs (68%; including 36% pre-treated patients) in a prospective phase-II trial of sequential FMC-plus-alemtuzumab by our study group [5]. However, an OS benefit after alemtuzumab consolidation over single-agent alemtuzumab (Table 1) was not accomplished.

In essence, chemotherapy is not recommended as a first-line treatment in T-PLL, unless there is well-documented severe intolerance towards alemtuzumab. It is rather an option in relapsed or primary alemtuzumab-refractory patients with the best clinical evidence for bendamustine. FMC is the most active first-line chemotherapy regimen, likely also to be active in a salvage situation [5, 26•]. Encouraging data on the nucleoside cladribine are discussed in “New rational-based approaches at conceptual stages and with first (pre)clinical evidence”.

### Alemtuzumab Remains the Current Benchmark

A milestone in the treatment of T-PLL was the implementation of alemtuzumab (Campath-1H). It is a fully-humanized IgG1 antibody targeting the surface CD52 antigen. Virtually all T-PLL express CD52 and at a higher density than in other T cell and B cell malignancies [27]. Engagement of CD52 by alemtuzumab induces antibody-dependent cytolysis, activation of the complement system, and possibly direct apoptosis [28, 29].

In treatment-naïve T-PLL, alemtuzumab induces ORRs of 75–92% (CRs in 48–81%), with no major difference between its use as a single substance or in combination with a conventional cytostatic. The progression-free survival (PFS) ranged from 7 to 12 months in these series [5, 10, 26•, 30, 31•]. These data by far surpass those of sole chemotherapy-based inductions. Importantly, alemtuzumab should be administered intravenously; the subcutaneous route is inferior in terms of response rates and freedom from disease as demonstrated in three independent studies [10, 26•, 32].

Alemtuzumab is generally well-tolerated [5, 10]. Initial infusion reactions are the most common side effects. Hematotoxicity of grades 3/4 occurs in 10–20% during the recommended 12-week period of 3 × 30 mg i.v./week [33, 34]. A *PJP* and HSV/CMV prophylaxis (and regular CMV monitoring) has to be implemented during treatment with this highly immunosuppressive agent [35, 36]. CMV reactivations occur in 19–52%, of which around 2/3 are clinically relevant [5, 26•]. EBV-positive B cell lymphomas were reported as rare events under alemtuzumab in the context of multiple sclerosis and in 2 T-PLL patients [37, 38].

The combination of alemtuzumab with the FMC chemo-regimen did not prolong PFS in two studies [5, 26•], despite a higher rate of BM-confirmed CRs when intravenous alemtuzumab followed 4 cycles of FMC. These studies rather emphasized that the added toxicity by such a chemo-component can compromise the administration of effective dosages of alemtuzumab. Therefore, alemtuzumab is currently recommended as a single-agent first-line treatment. In scenarios of insufficient responses to alemtuzumab, the nucleoside analogs fludarabine or pentostatin are the most frequent chemotherapeutics to be added to this antibody.

To accomplish long-term disease-free survival is the main challenge after alemtuzumab induction. Although > 80% of patients demonstrate initial remissions, in virtually all of them the disease recurs (at median after ≈ 12 months) in the absence of a consolidation by an allo-HSCT. There is no data that would justify an alemtuzumab maintenance after an induction therapy [26•]. At relapse, expression of CD52 should be reassessed (not by BM-histochemistry, which is prone to false-negative results) as ≈ 50% of patients respond to alemtuzumab re-treatment, although with a shorter PFS and OS [10]. A combination of alemtuzumab with pentostatin showed ORRs of 69% in a mixed cohort (5 treatment-naive and 8 previously treated T-PLL) [39]. Alemtuzumab relapsed/refractory (r/r) T-PLL is currently the main target population of individual exploratory concepts and trials. Attempts to improve primary alemtuzumab responses and their durations, e.g., by targeted agents such as JAK inhibitors, are currently underway (e.g., NCT03989466).

### Hematopoietic Stem Cell Transplantation

T-PLL patients, in which a CR can be achieved, need to be considered for allo-HSCT, the currently only treatment option to confer long-term disease control in T-PLL. In our experience, only 30–50% of T-PLL patients are eligible for an allo-HSCT, most commonly because of reduced performance status, relevant comorbidities, and age. A wash-out period between the last alemtuzumab administration and HSCT infusion is recommended to avoid related engraftment failures [40].

Retrospective studies showed the potential of allo-HSCT in inducing durable remissions [5, 41–46]. Therein, a 3–5-year post-HSCT survival was achieved in ≈ 20–30% of transplanted patients. Underlying reasons for these sobering results are high rates of early relapse (30–40%) and a considerable transplantation related mortality (TRM) of 30–40%.

A recently published prospective multi-center observational study emphasized the possibility of achieving a long-term disease-free survival in T-PLL patients after allo-HSCT (4-year PFS 30%, 4-year OS 42%) [47••]. By defining inclusion criteria in this registry, homogeneity of the analyzed cohort was accomplished and selection biases were reduced. While the overall relapse incidence was ≈ 38% after 4 years, this rate was significantly reduced in patients, who received conditioning regimens containing a total body irradiation of 6 Gy or more. Notably, an association of the time between alemtuzumab and allo-HSCT with disease control or non-relapse mortality, as previously described [41], was not observed. Furthermore, a graft-versus-leukemia (GvL) activity is described for T-PLL, as demonstrated by a reduction of minimal residual disease after immune modulations [48]. Overall, with the restrictions of sample size and study heterogeneity allowing no legitimate comparisons, outcomes in T-PLL patients after allo-HSCT seem to have improved within the last years (4-year OS 42% published in 2019 [47••] vs 2-year OS 21% published in 2012 [41], Table 2). Better patient selection (i.e. intent of allo-HSCT in first CR after alemtuzumab instead as a salvage attempt) and general advances in the allo-HSCT procedure may represent the main causes for such improvements.

**Table 2:** Summary of clinical studies that evaluated hematopoietic stem cell transplantation in T-PLL

Study design	*n*	Age [years]	Relapse rate [%]	TRM [%]	Pre-HSCT CR [%]	Post-HSCT CR [%]	PFS [mo]	OS	Reference
Summary of clinical studies on auto-HSCT in T-PLL									
Multi-center, retrospective	15	58	60	7	87	100	n.a.	52 mo	[42]
Summary of clinical studies on allo-HSCT in T-PLL									
Multicenter, retrospective	13	51	33	31	69	92	n.a.	33mo	[42]
CIBMTR registry, retrospective	21	54	39*	28*	n.a.	n.a.	5.1	11.2mo	[43]
EBMT registry, retrospective	41	51	41**	41**	27	n.a.	10	21%**	[41]
Multicenter, prospective	5	n.a.	n.a.	n.a.	n.a.	n.a.	n.a.	24.8mo	[5]
French Society of SCT, retrospective	27	53	47	31**	52	78	26**	36%**	[44]
Single-center, retrospective	11	56	21***	34***	91	91	15	56mo	[45]
EBMT registry, prospective (a)	37	56	38***	32***	62	n.a.	30***	42%***	[47••]
TRUMP registry, retrospective	20	54	69.6**	20.9*	30	n.a.	33.5%**	40%**	[46]

(a) Patients < 65 years, with progressive disease, with a mismatched unrelated donor or with cord blood were excluded

*at one year

**at three years

***at four years

EBMT: European Society for Blood and Marrow Transplantation; CIBMTR: Center for International Blood and Marrow Transplant Research; TRUMP: Transplant Registry Unified Management Program, Japan

Nevertheless, considering the still high relapse rates and above-average TRM, allo-HSCT in T-PLL requires profound optimizations: (i) robust strata that better identify who would benefit from an allo-HSCT, (ii) primary induction of more profound remissions without adding toxicity in patients that enter allo-HSCT, (iii) conditioning regimens that include specifically T-PLL-targeting components, (iv) better diagnostic and therapeutic tools to address residual T-PLL that causes post-transplant relapse, and (v) general strategies reducing the TRM and better directing the dichotomy of GvL vs GvHD.

When an allo-HSCT is not feasible (e.g., T-PLL patients without a donor), autologous HSCT (auto-HSCT) after high-dose chemotherapy can be considered. A post-induction auto-HSCT also achieved prolonged OS and PFS, but was not associated with long-term survival [42]. In addition, relapse rates are significantly higher, while TRM is lower compared to patients receiving an allo-HSCT. Table 2 provides a summary of studies that evaluated allo- and auto-HSCT in T-PLL.

## New Rational-based Approaches at Conceptual Stages and with First (Pre)Clinical Evidence

Given that cures are only accomplished in a very small subset of T-PLL and that for the majority of patients the prognosis is dismal, there is an urgent need for new substances, especially in alemtuzumab r/r disease. Recent in vitro drug screening approaches revealed new compound classes (e.g., p53 reactivators, epigenetic modulators, JAK inhibitors, and CDK inhibitors) as potentially active in T-PLL [13••, 17, 18••]. Of note, these studies did not involve therapeutic antibodies, did not address T-PLL in the context of (immune)milieu components, and did not systematically address synergistic relationships. Nevertheless, their findings of identified pathway dependencies align well with a recently advanced mechanistic disease concept of a (epi)genetically defined phenotype of inefficient checkpoint induction (e.g., repair, cell-cycle arrest, apoptosis) after DNA-damage and stress induction [13••].

Importantly, heterogeneous responses across T-PLL patients in vitro and in vivo are reported for these promising drug categories. This calls for better integration with molecular data towards models of individual sensitivity prediction [49]. In the following, we will focus on those substance classes and principles that have been evaluated for T-PLL specifically. Publications on such novel strategies are summarized in Table 3.

**Table 3:** Reference list summarizing the most important literature on new interventional strategies in T-PLL

Novel therapeutic approaches	
Strategy	Reference
Epigenetic modulation	[13••, 17, 18••, 52]
P53 reactivation	[13••, 18••]
Inhibition of constitutive JAK/STAT signaling	[17, 18••, 19, 20]
Antagonists of BCL2 family molecules	[17, 18••, 59•, 60, 61, 64, 65]
Inhibitors of cyclin-dependent kinases	[18••]
Modulation of immune cell synapses	[76]
CAR-T cell therapy	[79••]

### Epigenetic Modulation

Recent genomic profiling series revealed high frequencies of mutations of genes being essential in histone modifications, e.g., mutations of the histone methyltransferases *KMT2C*, *KMT2D*, *KMT5A*, and *EZH2* [12•, 13••, 16, 50]. In addition, the expression of genes encoding for components of epigenetic regulation is significantly altered in T-PLL [13••]. Because post-transcriptional modifications mediated by epigenetic regulators play an important role in DNA-damage repair via de-/acetylation of histones and of proteins like p53 or ATM [51], epigenetic dysregulations represent a targetable vulnerability in T-PLL.

In accordance with the high incidence of genomic alterations affecting epigenetic regulators in T-PLL, screening approaches identified inhibitors of HDACs as one of the most potent substance classes in T-PLL in vitro [13••, 17, 18••]. Importantly, HDAC inhibitors (vorinostat, belinostat, romidepsin) are FDA approved for the treatment of nodal and cutaneous peripheral T cell lymphomas (PTCL, CTCL). In a series of eight relapsed T-PLL patients, the combination of HDAC inhibitors with cladribine, a purine analog with epigenetic activity, enhanced the anti-leukemic activity of alemtuzumab and overcame previous treatment resistance towards this antibody as shown by responses in all treated patients (7 of 8 achieved a CR) [52]. Furthermore, an induction of CD30 expression after treatment with this combination was reported therein. Although our laboratory and others were not able to induce CD30 upon exposure to this combination (data not shown), these authors report that treatment with the anti-CD30 immunotoxin brentuximab vedotin led to a reduction of alemtuzumab refractory skin lesions after such epigenetic priming in two intensively pretreated T-PLL patients [52].

Other epigenetic approaches focus on the combination of HDAC inhibition with DNA-damage induction. We described a rational-based marked in vitro synergy in T-PLL by the pan-HDAC inhibitor panobinostat in combination with bendamustine [13••]. Moreover, tinostamustine (EDO-S101), a fusion molecule of vorinostat covalently linked to bendamustine, was granted FDA fast-track approval for T-PLL based on our pre-clinical data [53]. This novel deacetylating alkylator displayed promising in vitro and in-vivo anti-T-PLL activity and is likely to be investigated further in T-PLL patients and in other hematologic and solid-tumor entities.

### Strategies of p53 Reactivation

In contrast to many other tumors, the *TP53* gene in T-PLL is rarely the target of deletions or mutations [13••]. Instead, we demonstrated the marked inability of the T-PLL cell to evoke p53 activation due to deficient upstream signals through hypomorphic ATM [13••]. This led to a concept of functionally intact p53 to be arrested in an inactive state bound to its inhibitor mouse double minute 2 (MDM2) and being largely deacetylated [16]. We reasoned from this that deacetylase inhibitors (i.e., HDAC inhibitors) and MDM2/4 antagonists would act as anti-leukemic p53 derepressors in T-PLL. Indeed, treatment of T-PLL cells with the MDM2 inhibitor idasanutlin restored the functional capacity of p53 as demonstrated by its phosphorylation and acetylation, which was even more prominent in combination with the pan-HDAC inhibitor panobinostat [13••]. Moreover, in an independent screen of 301 drugs, the p53 reactivator Prima-1-Met was the second most efficient substance inducing cell death of T-PLL cells in vitro [18••]. In addition, the MDM2 antagonists serdemetan and nutlin-3 were among the 20 most potent drugs in this study. Another ex vivo drug profiling effort further underlined the synergism between p53 reactivation and HDAC inhibition [16]. Importantly, the presented MDM2 antagonists showed selectivity in killing T-PLL cells with nearly no cell death in normal T cells. MDM2 antagonists are currently tested for advanced solid and hematologic tumors in early-stage trials, however, their clinical applicability in T-PLL still has to be evaluated.

Of note, the efficiency of poly ADP-ribose polymerase (PARP) inhibition was reasoned based on the assumption of a synthetic lethal relationship with the mutated and deleted *ATM* recurrently found in T-PLL (> 85%). However, such a hyper-sensitivity was not observed [13••].

### Inhibition of Constitutive JAK/STAT Signaling

Gain-of-function mutations in *JAK* and *STAT* genes have been described recently to be highly frequent in T-PLL, varying from 36% to 76%, although often found as sub-clonal lesions [15•]. This makes the JAK/STAT pathway, a cytokine-triggered mediator of proliferation, differentiation, and migration of T cells, an interesting target [54]. Predominantly, *JAK1*, *JAK3*, and *STAT5B* are affected, accompanied by a high prevalence of genomic losses of negative regulators (71%) [15•]. Such genomically determined JAK/STAT activation in 90% of T-PLL explains the elevated STAT5b phosphorylation we observed in virtually all cases [13••, 15•]. In the mentioned sensitivity screen by Andersson et al., JAK inhibitors were among the 25 most effective substances in inducing cell death in T-PLL [18••]. The frequently occurring *STAT5B* N642H mutation even predicted resistance towards JAK inhibition. These results were supported in another drug screen of a large panel of blood cancers, in which T-PLL showed the highest sensitivity towards JAK inhibitors [17]. JAK inhibitors are currently approved in autoimmune conditions and GvHD, while inhibitors of STAT proteins are still under pre-clinical development [55]. First case reports on the clinical use of JAK inhibitors in T-PLL presented moderate activity of tofacitinib (inhibition of JAK2/3) and ruxolitinib (inhibition of JAK1/2) [19, 20]. A phase-I trial investigating the safety and tolerability of the JAK1 inhibitor itacitinib in combination with alemtuzumab in T-PLL is currently commencing recruitment (NCT03989466). Therapeutic approaches targeting JAK/STAT signaling have to be expanded, including the development of new, SH2-selective or N-domain targeting STAT5 inhibitors [56].

### Antagonists of BCL2 Family Molecules

P53 mediates apoptosis through direct effects at the mitochondrial membrane as well as through transcriptional activation of pro-apoptotic B cell lymphoma 2 (BCL2) family molecules. An equilibrium of the concentrations and affinities of pro- and anti-apoptotic BCL2 proteins (both multidomain), as well as BH3-only proteins hereby regulates apoptosis induction. Interaction between pro- and anti-apoptotic molecules and between the pro-apoptotic proteins and the effectors BCL2-associated X protein (BAX) and BCL2 homologous antagonist killer (Bak) takes place at the BH3 domain, which is therefore essential for apoptosis induction by modulation of the mitochondrial outer membrane permeability [57]. As p53 activation is deficient in T-PLL (see “Strategies of p53 Reactivation”), inducing apoptosis downstream of p53 by targeting the BH3 domain of anti-apoptotic BCL2 family proteins with BH3 mimetics is a reasonable strategy [16]. Mutations in *BCL2* family genes are not described in T-PLL [12•, 13••, 58].

Ex vivo screens revealed high anti-leukemic activity of the BCL2 inhibitors navitoclax (targets BCL2 and BCL-XL) and venetoclax (BCL2 only) in T-PLL [17, 18••]. It is still not finally resolved whether the responses to venetoclax correlate with the expression of BCL2, or with the expression of other anti-apoptotic family members such as myeloid cell leukemia 1 (MCL1) or B cell lymphoma XL (BCL-XL)(own data, not shown and [59•]). In comparison to CLL, BCL2 dependence appears lower and cells were less primed towards apoptosis in T-PLL, while MCL1 dependences are comparable, as revealed by first BH3-profiling studies [60].

First clinical case reports showed partial responses (PRs) after treatment with single-agent venetoclax in 2 r/r T-PLL patients [59•]. The first patients responded rapidly but developed a tumor lysis syndrome during dose increments, while the ramp-up was completed in the second patient without any tumor lysis syndrome and a PR was achieved for 131 days. Induction of BCL2 and BCL-XL protein expression was observed in these patients upon venetoclax treatment, potentially explaining modes of venetoclax resistance.

Aiming at identifying potentially synergistic partners, a combination screen identified ibrutinib (inhibitor of IL-2 inducible T cell kinase, ITK) as a suitable co-treatment. This was underlined by an increase of BCL2 dependence after ibrutinib priming, the latter having no effect on T-PLL cell viability [61]. This corroborates data by us and others, in which single-agent ITK inhibitors led to a diminished activation upon T cell receptor (TCR) stimulation, but did not affect viability of T-PLL cells [62, 63]. Two r/r T-PLL patients treated with the combination of venetoclax and ibrutinib showed substantial clinical responses [61]. Based on these pilot data, an international study evaluating the efficiency of venetoclax plus ibrutinib in alemtuzumab r/r T-PLL was initiated and is currently recruiting (VIT trial, NCT03873493).

BCL2 dependence can obviously also be enhanced by co-treatment with JAK/STAT or HDAC inhibitors, leading to a pronounced in vitro anti-T-PLL effect of venetoclax [60]. Combination of venetoclax with the purine analog pentostatin was tested in one r/r T-PLL case, resulting in a CR [64]. Besides targeting BCL2, inhibition of the anti-apoptotic MCL1 represents another strategy to be further investigated in T-PLL [65].

Future research has to identify markers that detect the likely considerable number of T-PLL that are insensitive to BCL2 inhibition, has to elucidate mechanisms of resistance towards BH3 mimetics and how to overcome them, and has to determine the most ideal combination partners of this compound class.

### Inhibitors of Cyclin-dependent Kinases

In the before-mentioned drug screen of 301 compounds, the CDK inhibitor SNS-032 was the top-scoring substance [18••]. SNS-032 particularly inhibits CDK-2, -7, and -9. Notably, sensitivity of T-PLL cases towards SNS-032 correlated with high MYC expression [18••]. Downregulation of MCL1 is described as one potential mechanism through which inhibitors of CDK9 work, leading to the hypothesis of a good synergism between specific CDK9 inhibitors and BH3 mimetics, which has to be evaluated in future research [66].

### Potential Approaches Adopted from Other T Cell Lymphomas

Molecules under investigation for the treatment of other T cell lymphomas will likely also influence the field of T-PLL [67]. Important to mention is the inhibition of the phosphoinositide 3-kinase (PI3K, e.g., via Copanlisib, Duvelisib) or combined JAK/STAT-SYK inhibitors (e.g., Cerdulatinib) [67–69]. Ex vivo, blocking of PI3K led to a reduced growth of primary T-PLL cells [6]. Other strategies focus on targeting chemokine receptors via antibodies. Mogamulizumab (anti-C-C chemokine receptor type 4) currently enters the treatment of CTCL [70].

At the epigenetic level, besides HDAC inhibition, targeting of EZH1/2 appears as a reasonable strategy, as suggested by the high frequency of mutations of this histone methyltransferase in T-PLL [12•, 13••, 16, 50]. EZH2, which plays a role in T cell differentiation and malignant transformation, was previously described as highly expressed across most PTCL [71, 72]. GSK2816126, which inhibits wildtype and mutated EZH2, is currently tested in advanced solid and hematologic tumors, although with signals of considerable side effects [73].

### Modulation of Immune Cell Synapses

Inhibitors of the T cell immune-checkpoints are being tested in PTCL [67]. However, there are several notes of caution to be stressed with respect to uncritical applications to any T cell tumor. T-PLL shows marked normal-T cell penia and tumor cells have drastically down-regulated checkpoint molecules, including PDL1, without knowing whether this is true for the hardly detectable non-malignant (effector T cells) [13••]. Furthermore, PD1 is a potent tumor-suppressor, and its blockade may lead to tumor progression as experimentally implicated [74]. Importantly, clinical hyper-progressions have already been described in PTCL after nivolumab [75].

Another approach of immunogenic cell death induction is to exploit the interaction of CD47 with its ligand signal regulatory protein α (SiRPα). By targeting tumor-cell CD47 through a decoy receptor (SiRPαFc), reverting the “do-not-eat-me” signal to bystander macrophages, a significant reduction of tumor load was observed in various hematologic malignancies, i.e., CTCL [76]. Perturbation of NK-cell tolerance, e.g., by anti-KIR3DL2 antibodies, is a further strategy of proven activity in other T cell tumors [76, 77]. The clinical utility of these concepts in T-PLL should be part of future research.

### Outlook for CAR-T Cell Therapy

The emerging field of chimeric antigen receptor T cells (CAR-T cells) also raised interest in the field of T cell malignancies [78]. Antigens that are unique to the T cell malignancies are rare, an exception might be CD30. Therefore, CAR-T cell-based targeting of pan-T cell antigens would lead to severe T cell immunosuppression as well as CAR-T cell fratricide. An elegant study took advantage of the TCR beta-chain constant (TRBC) locus clonality. While normal T cell populations are a mixture of TRBC1 and TRBC2 positive cells, malignant Tcells only express one beta-chain. First in vitro data showed that CAR-T cells, selectively targeting the TRBC of the malignant clone, specifically killed T-PLL cells, while sparing healthy T cells expressing a different TRBC [79••]. Another possible target for personalized CAR-T cells is the complementarity-determining region 3 (CDR3), which is the hypervariable region of the TCR being responsible for binding of the antigen. Data on anti-CDR3 CAR-T cells have not been reported in T-PLL [80].

## Conclusions

T-PLL remains a highly problematic disease with poor outcome data and very limited therapeutic options. Induction by infusional alemtuzumab followed by a consolidating allo-HSCT in the 30–50% eligible patients leads to a long-term survival in a < 50% fraction of transplanted patients, resulting in 5–10% of all T-PLL patients that would have such a benefit from this approach. The modalities in alemtuzumab r/r disease are of lesser efficacy and the responses are short-lived.

This review, however, shall stimulate optimism among physicians, researchers, and patients. The field of T-PLL has never been that active before. Academia and pharmaceutical companies gain increasing interest in T cell tumors, including T-PLL, for which we still lack approved drugs. We have arrived at a much better understanding of the molecular make-up of T-PLL and have identified key vulnerabilities [13••]. Consequently, novel therapeutic approaches are on the horizon, mainly represented by epigenetic modulators, p53 reactivators, JAK/STAT inhibitors, and BH3 mimetics, with the latter being closest to systematic clinical testing (Fig. 1). These will likely be supplemented by those that prove to be successful in other T cell malignancies. They will include in addition to repurposed cytostatics, so-called small molecules, novel antibodies, immune-synapse reshaping agents, or CAR-T-cells.

We are looking forward to more-than-ever concerted efforts (i) to identify novel vulnerabilities in T-PLL, (ii) to describe optimal drug synergisms (including those with alemtuzumab or allo-HSCT protocols), (iii) to develop prediction tools for individual (pre)clinical responses, (iv) to devise strategies in dealing with resistance, and (v) to implement this in international clinical trials, overall to substantially improve the outcomes of T-PLL. Researchers in this orphan disease should also understand themselves as advocates for the multi-faceted needs of T-PLL patients, e.g., access to specialists, to off-label options, and to trial sites as well as in the promotion of fast-track drug approvals.
